# Supplementary material for: Biocomposite thermoplastic polyurethanes containing evolved bacterial spores as living fillers to facilitate polymer disintegration
Source: Nat Commun. 2024 Apr 30;15:3338. doi: 10.1038/s41467-024-47132-8 (PMC11061138; doi:10.1038/s41467-024-47132-8)
Supplement: Supplementary file 1 — Supplementary Information [file 41467_2024_47132_MOESM1_ESM.pdf]

Supplementary Information for

Biocomposite Thermoplastic Polyurethanes Containing Evolved Bacterial Spores as Living Fillers to Facilitate Polymer Disintegration

Han Sol Kim[1,a], Myung Hyun Noh[2,3,a], Evan M. White[4], Michael V. Kandefer[4], Austin F. Wright[4], Debika Datta[1], Hyun Gyu Lim[2], Ethan Smiggs[2], Jason J. Locklin[4], Md Arifur Rahman[5*], Adam M. Feist[2,6,*], Jonathan K. Pokorski[1,7,*]

[1]*Department of NanoEngineering, University of California San Diego, 9500 Gilman Dr., La Jolla, CA 92093, USA*

[2]*Department of Bioengineering, University of California San Diego, 9500 Gilman Dr., La Jolla, CA 92093, USA*

[3]*Research Center for Bio-based Chemistry, Korea Research Institute of Chemical Technology (KRICT), 406-30 Jongga-ro, Ulsan, 44429, Republic of Korea*

[4]*New Materials Institute, University of Georgia, Athens, GA 30602, USA*

[5]*Thermoplastic Polyurethane Research, BASF Corporation, 1609 Biddle Ave., Wyandotte, MI 48192, USA*

[6]*The Novo Nordisk Foundation Center for Biosustainability, Technical University of Denmark, Building 220, Kemitorvet, 2800 Kgs. Lyngby, Denmark*

[7]*Institute for Materials Discovery and Design, University of California San Diego, 9500 Gilman Dr., La Jolla, CA, 92093, USA*

[a]These authors contributed equally to the work

*Co-correspondence to: md-arifur.rahman@basf.com, afeist@ucsd.edu, jpokorski@ucsd.edu

Table of Contents

Supplementary Fig. 1. TPU assimilation activity of ATCC 6633 strain 1

Supplementary Fig. 2. Photograph of biocomposite TPU extrudate 2

Supplementary Fig. 3. SEM images of lyophilized spores 3

Supplementary Fig. 4. UV-Vis spectra of spores extracted from biocomposite TPU 4

Supplementary Fig. 5. Shear stress during hot melt extrusion.. 5

Supplementary Fig. 6. Metabolic activity of ATCC 6633 WT and HST strains 6

Supplementary Fig. 7. X-ray microscopy 7

Supplementary Fig. 8. Toughness comparison... 8

Supplementary Fig. 9. Stress versus strain curves 9

Supplementary Fig. 10. Fitting tensile stress using Pukánszky model 10

Supplementary Fig. 11. Water contact angle analysis 11

Supplementary Fig. 12. Gel permeation chromatography 12

Supplementary Fig. 13. Attenuated total reflectance-Fourier transform infrared spectroscopy 13

Supplementary Fig. 14. Spore germination on compost extract gels 14

Supplementary Fig. 15. Gravimetric analysis of TPU degradation in untreated compost ... 16

Supplementary Fig. 16. Additional CLSM images 18

Supplementary Fig. 17. Tensile properties of commercial and biodegradable TPUs..... 19

Supplementary Fig. 18. Biocomposite TPU fabrication using benchtop twin screw extruder 20

Supplementary Fig. 19. Spore viability and maximum shear stress at different screw speed 21

Supplementary Fig. 20. Respirometry analysis of TPU degrdation in untreated compost .. 22

Supplementary Table 1. CFU assay of untreated and autoclaved compost 24

Supplementary Table 2. Elemental analysis of compost 25

Supplementary Table 3. Carbon and nitrogen content of test materials for respirometry 26

Supplementary Note 1. Spore germination on compost extract gels 15

Supplementary Note 2. Disintegration of biocomposite TPU in untreated compost 17

Supplementary Note 3. Respirometry analysis of TPU degradation in untreated compost . 23

Supplementary References.. 27

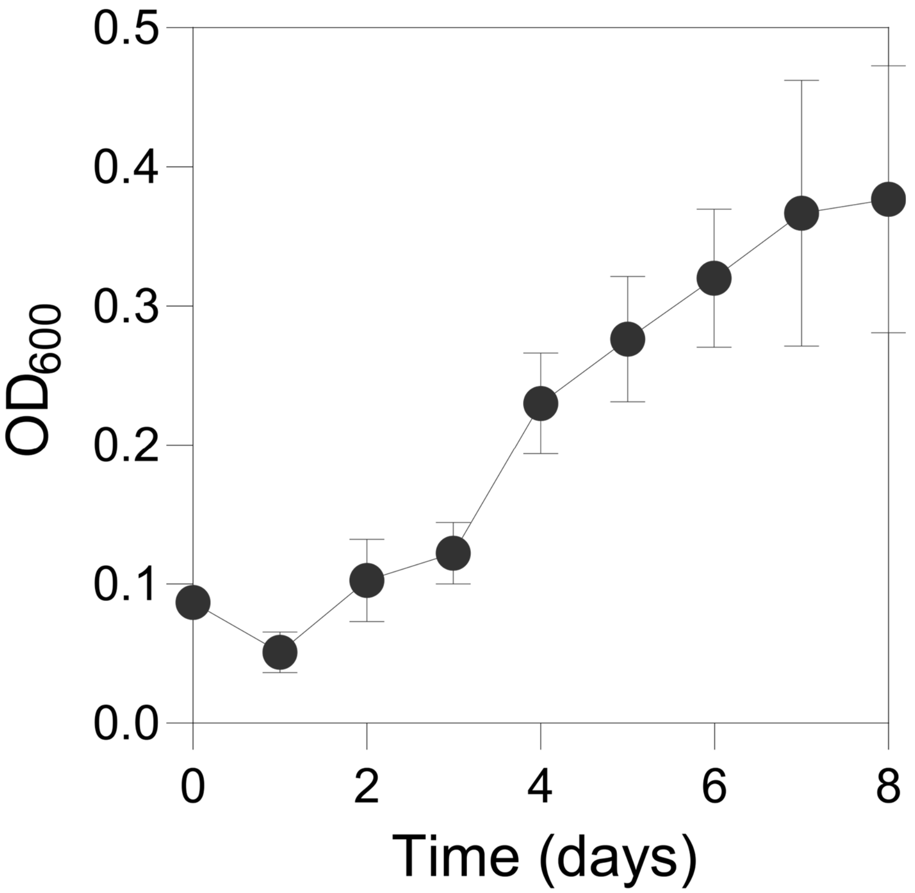

Supplementary Fig. 1. TPU assimilation activity of ATCC 6633 strain. Growth of *B. subtilis* ATCC 6633 strain (OD_{600}) by the assimilation of polyester-based TPU as a sole carbon source. TPU assimilation activity was determined by cell growth under M9 minimal medium supplemented with 10 g/L TPU powder (M9 + TPU) at 37 °C under 250 rpm shaking. Data are presented as mean values ± standard deviations from three independent experiments. Source data are provided as a Source Data file.

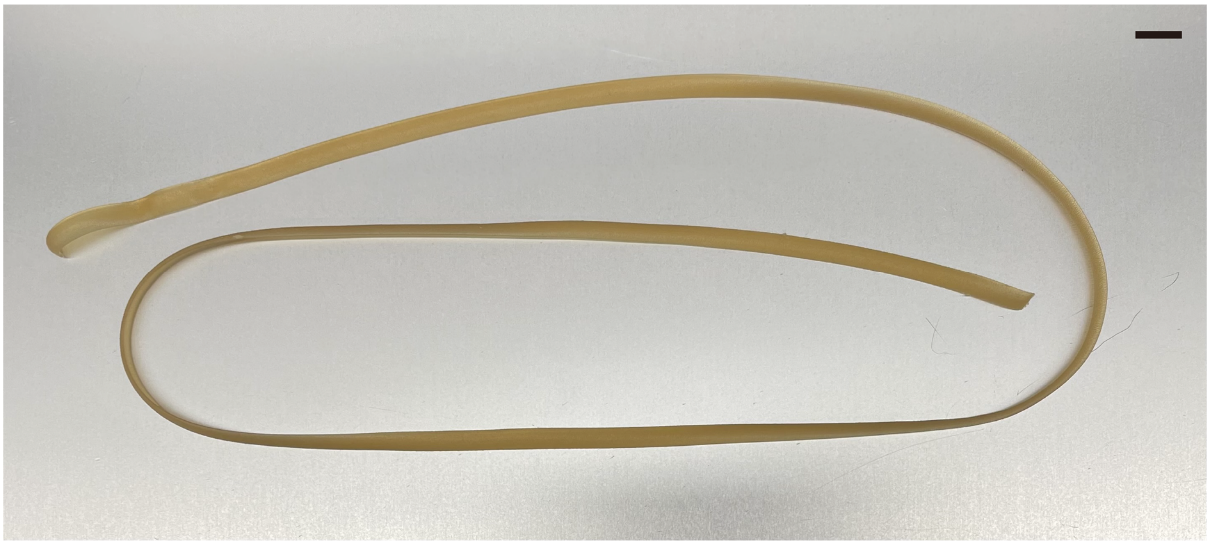

Supplementary Fig. 2. Photograph of biocomposite TPU extrudate. Full scale image of extruded BC TPU^{HST} with 0.8 w/w% spore loading (scale bar: 10 mm). The width and thickness of the extrudate was determined by the geometry of the slit die (5.0 mm x 0.7 mm). The final length of extrudate depended on the extrusion time and generally resulted in ~750 mm.

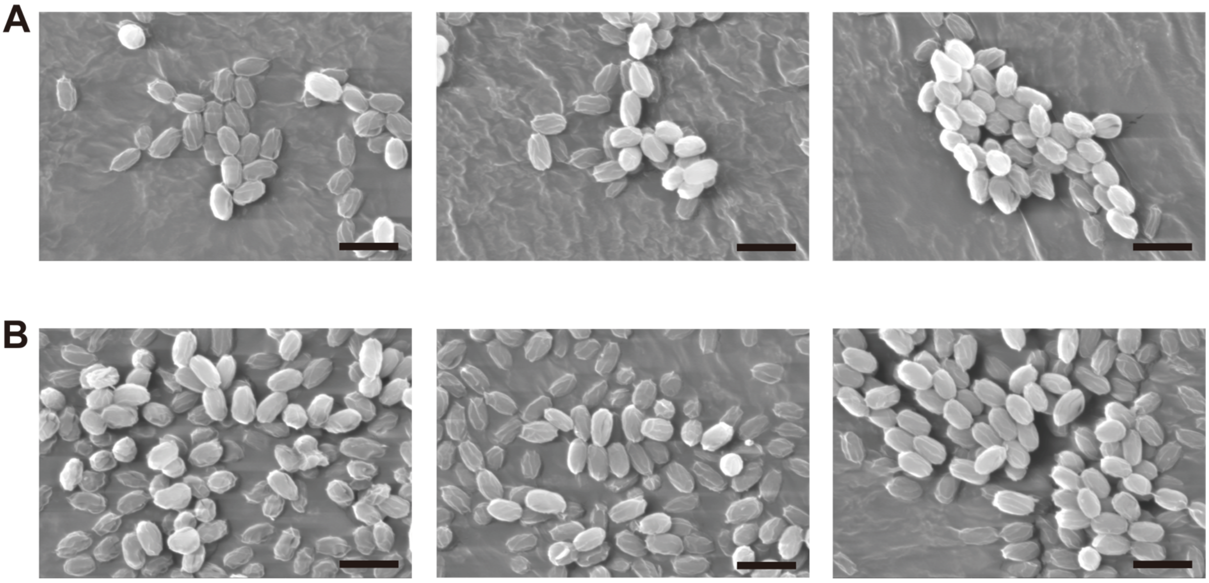

Supplementary Fig. 3. SEM images of lyophilized spores. Scanning electron microscope images of lyophilized WT (A) and HST (B) spores before the incorporation into TPU (10k magnification). Each panel represents an individual visualization site. Scale bars are 2 μm.

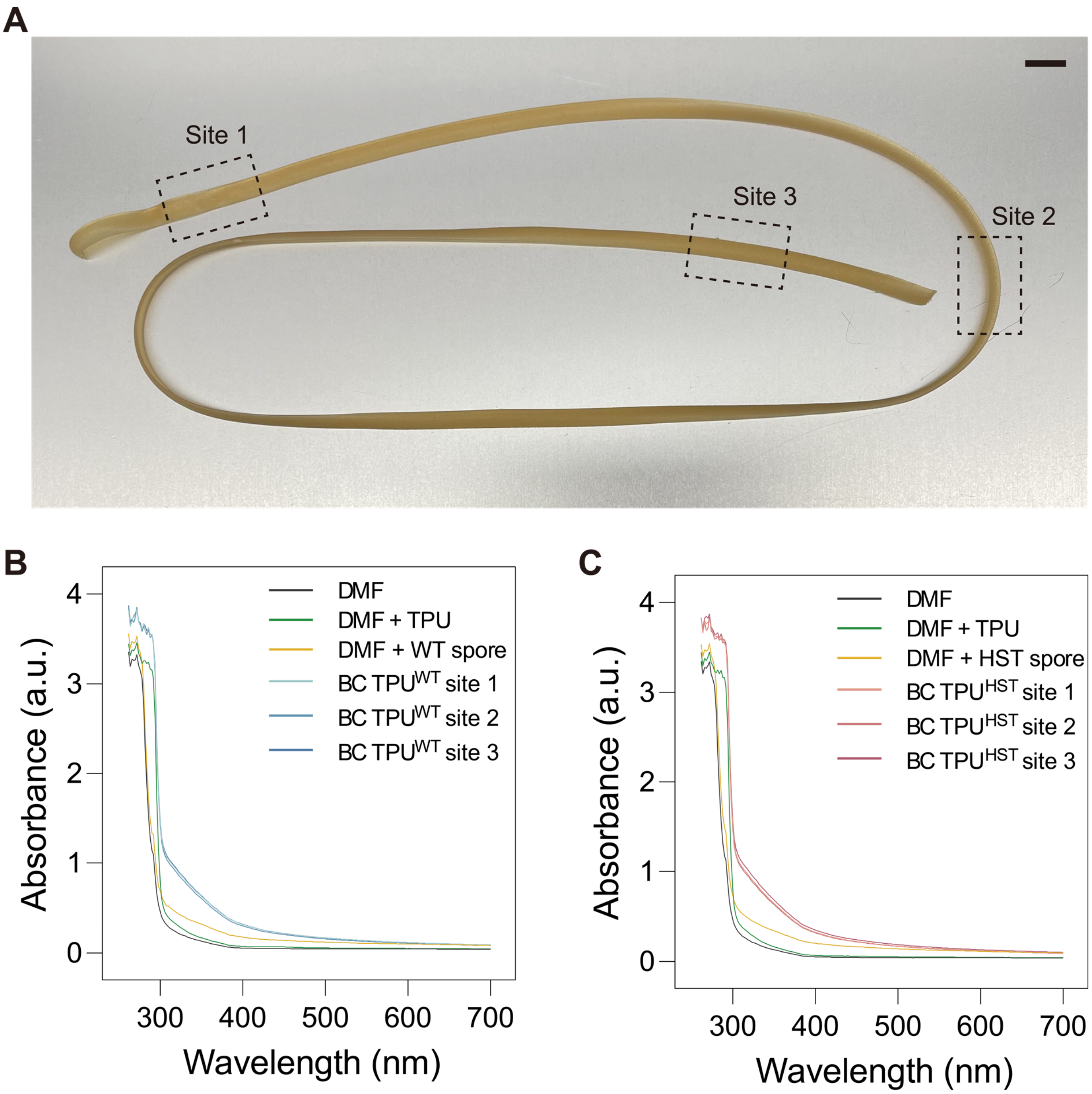

Supplementary Fig. 4. UV-Vis spectra of spores extracted from biocomposite TPU. (A) Exemplary sampling sites from TPU extrudate for the spectrophotometry (scale bar: 10 mm). UV-Vis spectra of BC TPUs with 0.8 w/w% WT (B) or HST (C) spores and their corresponding controls dissolved/suspended in DMF. Final concentrations of TPU and spore in DMF were 9.92 mg/mL and 0.08 mg/mL, respectively. TPU showed absorbance at ~300 nm, while spores absorbed a broad range of UV-Vis after 300 nm. BC TPUs showed characteristic absorbance patterns from both TPU and spore. Source data are provided as a Source Data file.

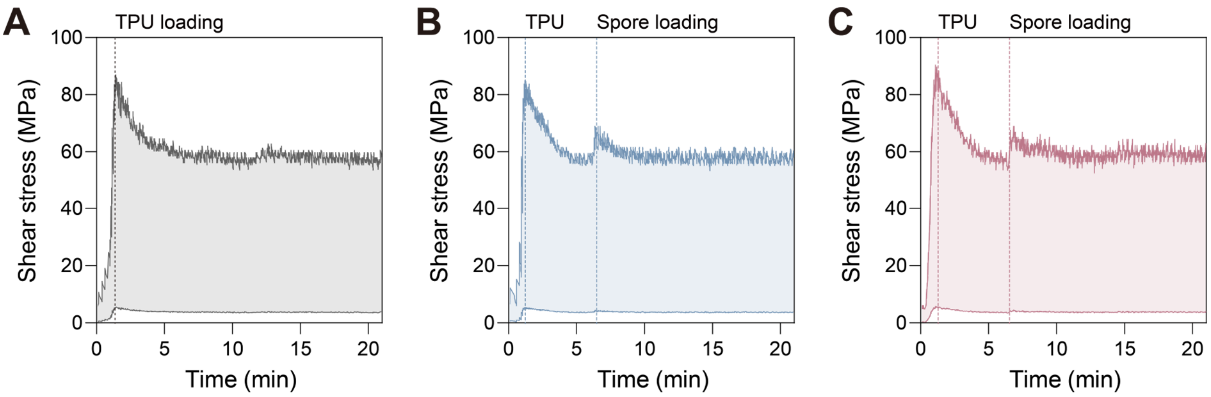

Supplementary Fig. 5. Shear stress during hot melt extrusion. Shear stress during melt processing of TPU (A), BC TPU^{WT} (B) and BC TPU^{HST} (C). Spore loadings of biocomposite TPUs were 0.8 w/w%. Spores were added after 5 min of TPU melting and equilibration in the twin screw extruder for biocomposite TPU fabrication. Source data are provided as a Source Data file.

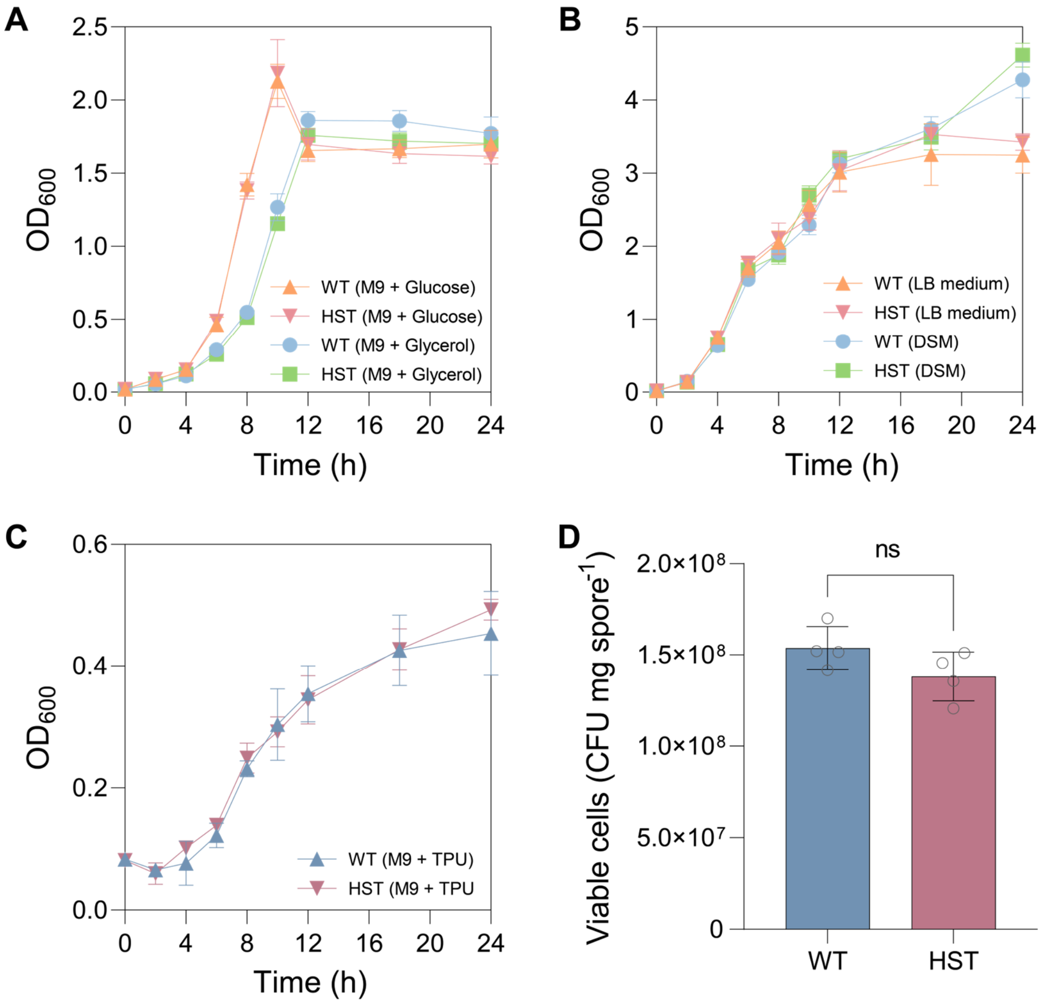

Supplementary Fig. 6. Metabolic activity of ATCC 6633 WT and HST strains. Cell growth profiles of ATCC 6633 WT and HST strains under M9 minimal medium supplemented with general carbon sources such as 4 g/L glucose (M9 + Glucose) or 4 g/L glycerol (M9 + Glycerol) (A) and complex media such as LB or DSM (B) at 37 °C under 250 rpm shaking. (C) TPU assimilation activity determined by cell growth under M9 minimal medium supplemented with 10 g/L TPU powder (M9 + TPU) as the sole nutrient source at 37 °C under 250 rpm shaking. (D) Spore viability of ATCC 6633 WT and HST strains. A two-sided Welch's t-test was used for statistical analysis (n = 4 per group; ns: not significant). Data are presented as mean values ± standard deviations from four independent experiments. Source data and detailed statistical analysis results are provided as a Source Data file.

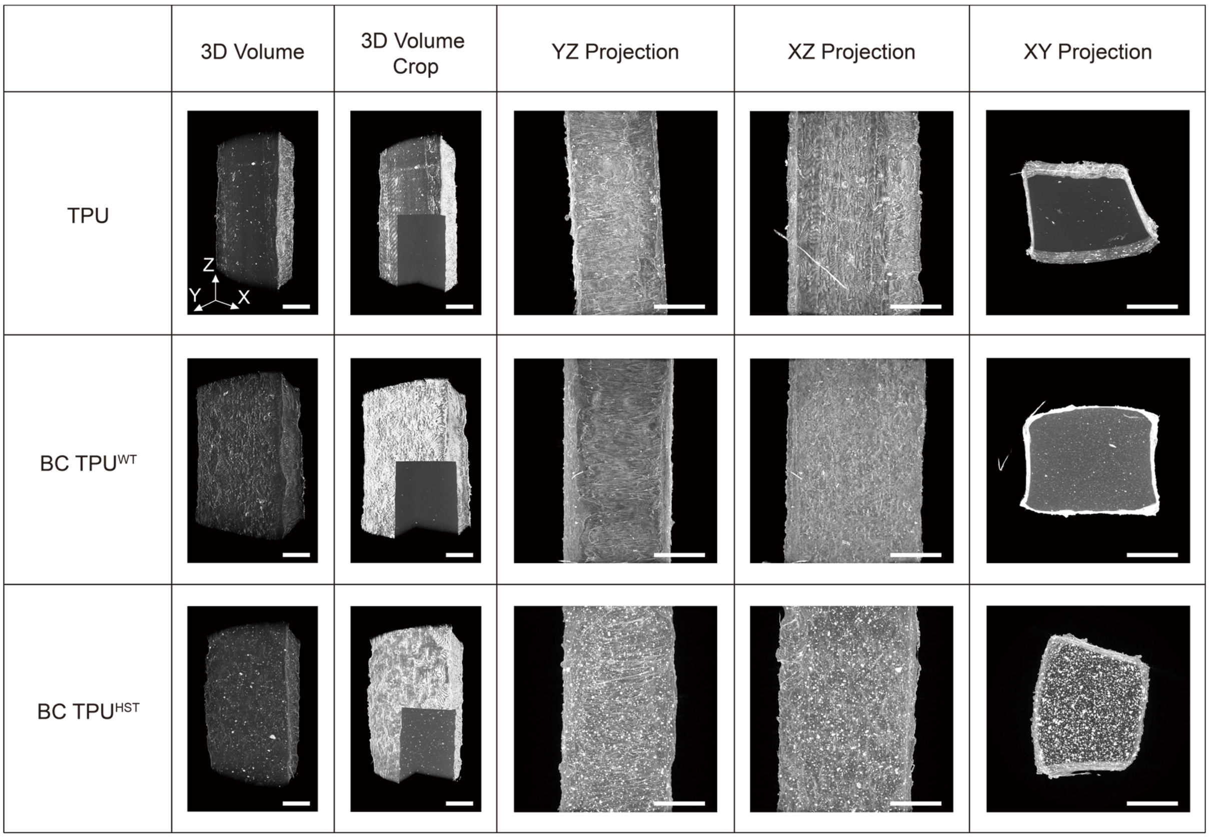

Supplementary Fig. 7. X-ray microscopy. MicroCT images of TPU, BC TPU^{WT} and BC TPU^{HST} were obtained using XRM (scale bars: 500 μm). Spore loadings of biocomposite TPUs were 0.8 w/w%. Specimens for XRM analysis prepared at ~1 x 1 x 10 mm^3 (X x Y x Z) dimension. 3D scanning was conducted by rotating the specimens in the Z axis. Since X and Y dimensions of each specimen were smaller than the scanning frame, the rough surface of the specimen was visualized in MicroCT images except XY projection.

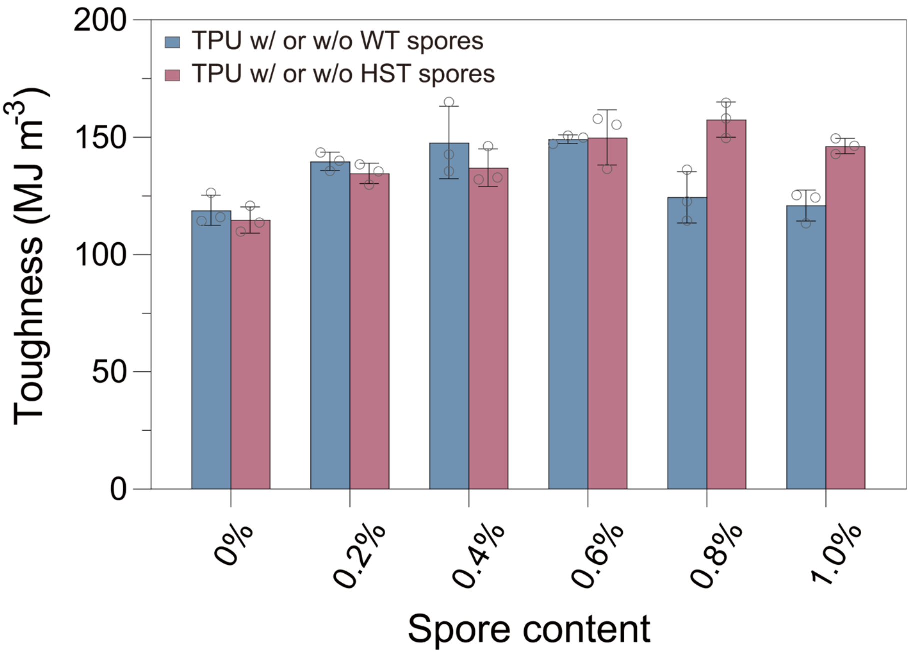

Supplementary Fig. 8. Toughness comparison. Toughness of biocomposite TPUs with WT and HST spores and their corresponding baseline TPUs combined in one graph. Source data are provided as a Source Data file.

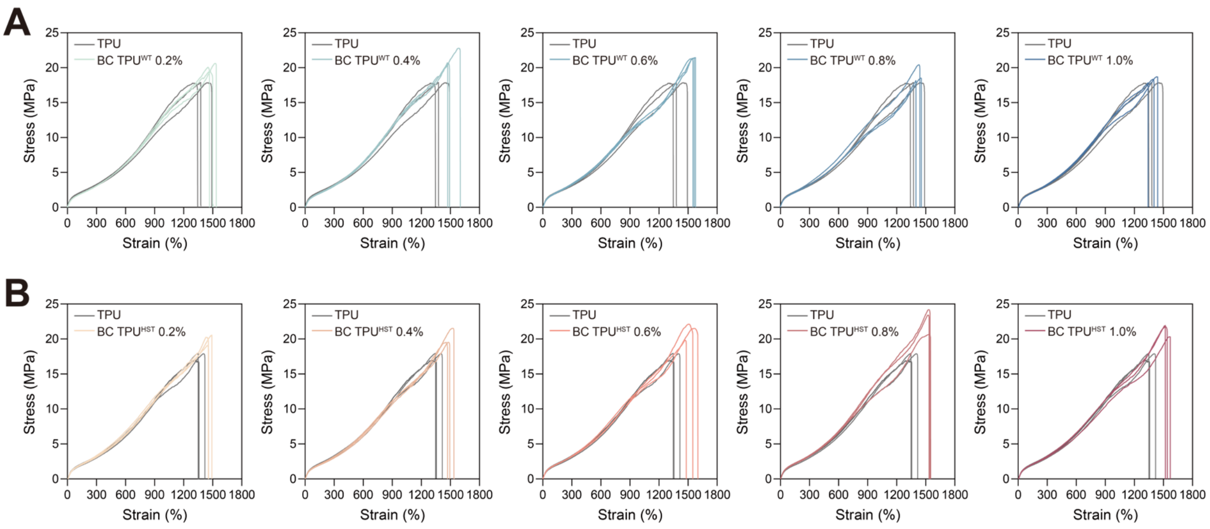

Supplementary Fig. 9. Stress versus strain curves. Stress versus strain curves obtained by the tensile testing of BC TPU^{WT} (A) and BC TPU^{HST} (B) with varying spore contents. Toughness was calculated from the area under the curve. Ultimate tensile stress was obtained by the Y peak, while the elongation at break was the strain at the moment of fracture. Young's modulus was calculated from the slope in the initial range. Source data are provided as a Source Data file.

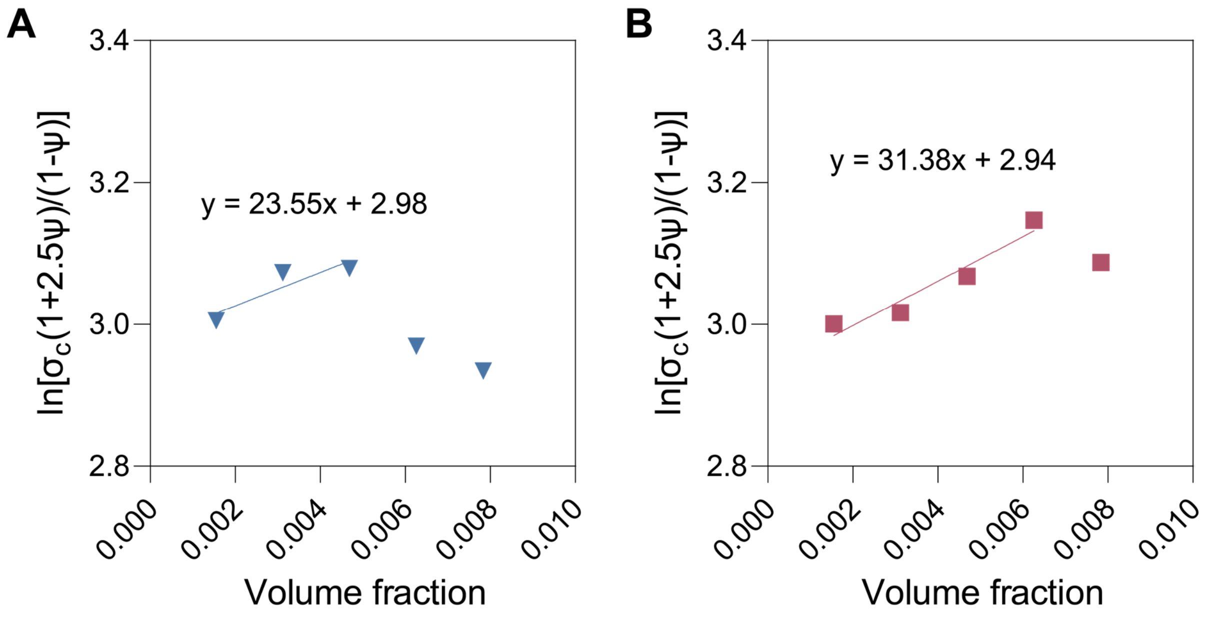

Supplementary Fig. 10. Fitting tensile stress using Pukánszky model. B values of BC TPU^{WT} (A) and BC TPU^{HST} (B) were calculated based on the Pukánszky model. Source data are provided as a Source Data file.

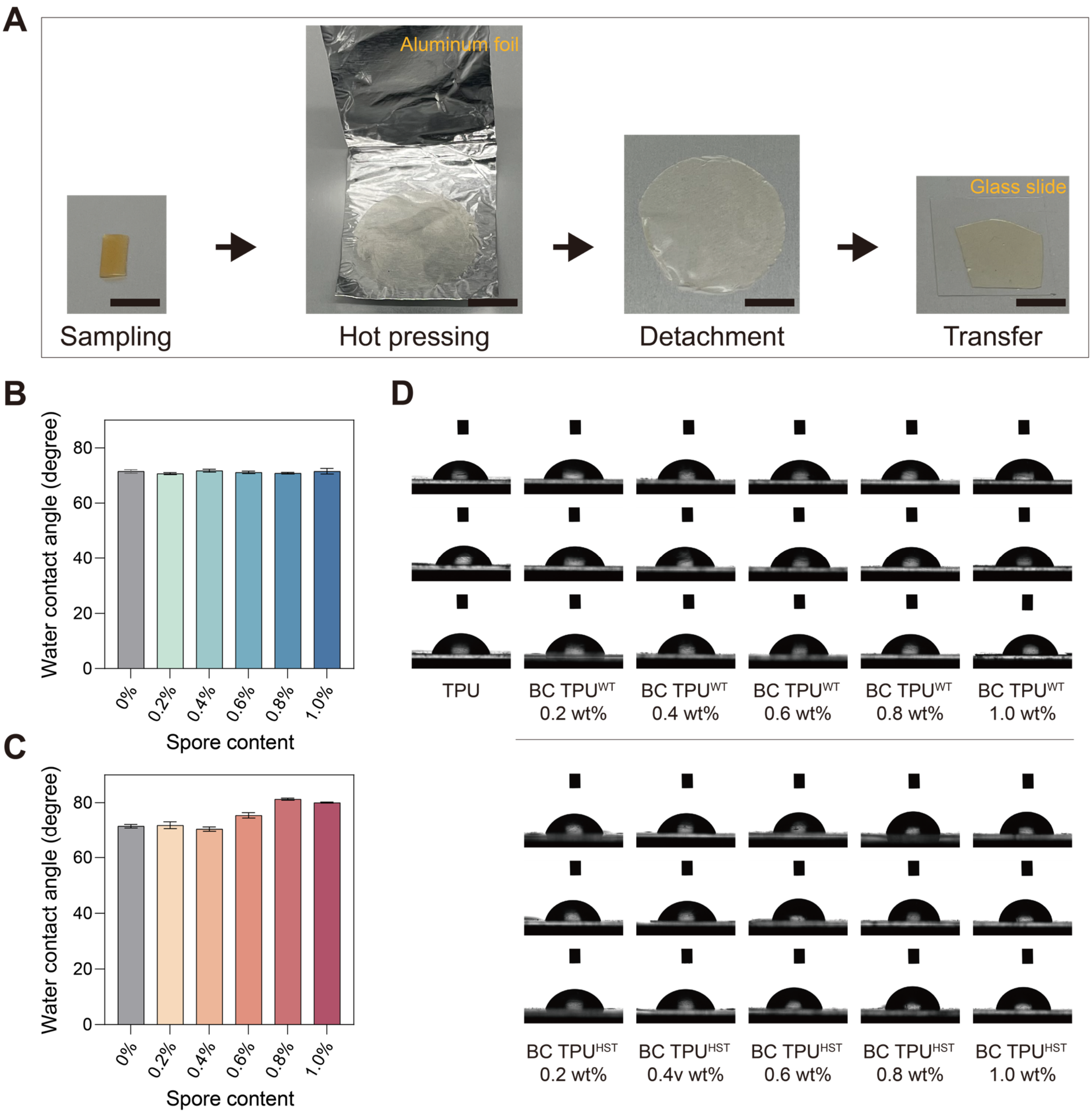

Supplementary Fig. 11. Water contact angle analysis. (A) Sample preparation for water contact angle analysis by using hot pressing (scale bar: 10 mm). Water contact angle of BC TPU^{WT} (B) and BC TPU^{HST} (C) with 0-1.0 w/w% spore loading. Data are presented as mean values ± standard deviations from three independent experiments. (D) Water contact angles were obtained by analyzing the photographs of water droplets on flattened BC TPU^{WT} and BC TPU^{HST}. Source data are provided as a Source Data file.

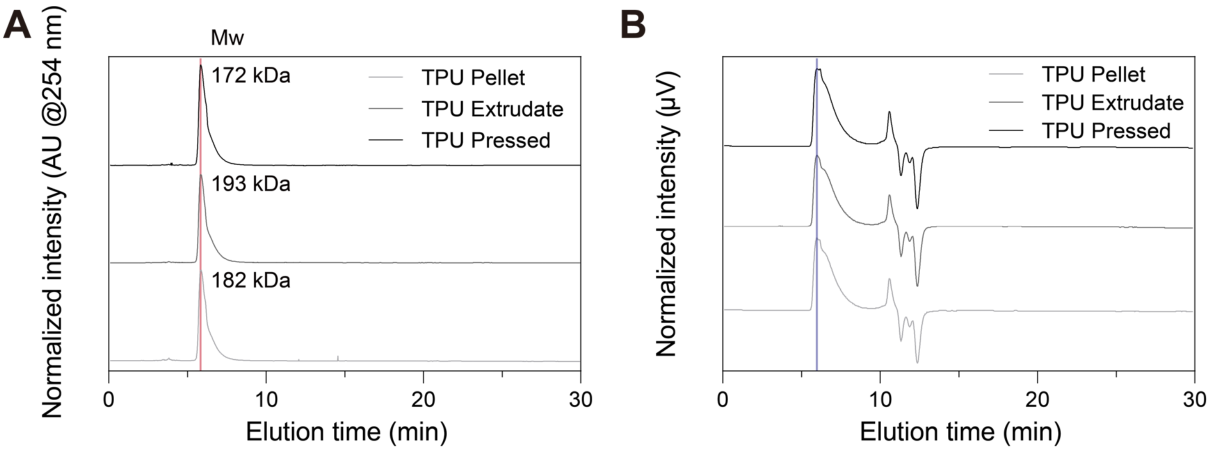

Supplementary Fig. 12. Gel permeation chromatography. Gel permeation chromatograms of TPU before and after hot melt extrusion and hot pressing obtained by using ultraviolet (A) and refractive index (B) detectors. The molecular weight of TPU remained similar post extrusion or pressing. Noise after 10 min elution time determined by refractive index detector are void peaks as different grades of THF (ACS reagent grade and HPLC grade) were used for dissolving TPU. Source data are provided as a Source Data file.

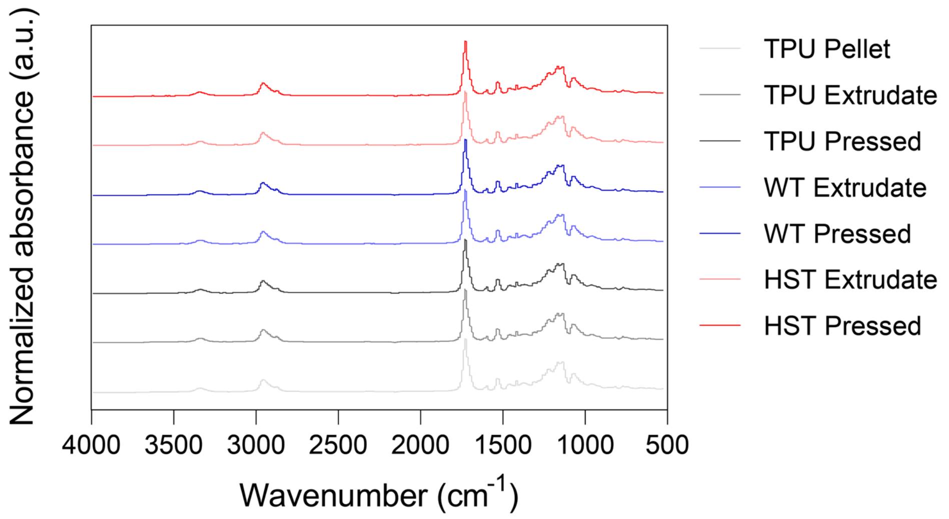

Supplementary Fig. 13. Attenuated total reflectance-Fourier transform infrared spectroscopy. Attenuated total reflectance-Fourier transform infrared spectroscopy of TPU and BC TPUs before and after hot melt extrusion or hot pressing. BC TPUs contained 0.8 w/w% spores. Source data are provided as a Source Data file.

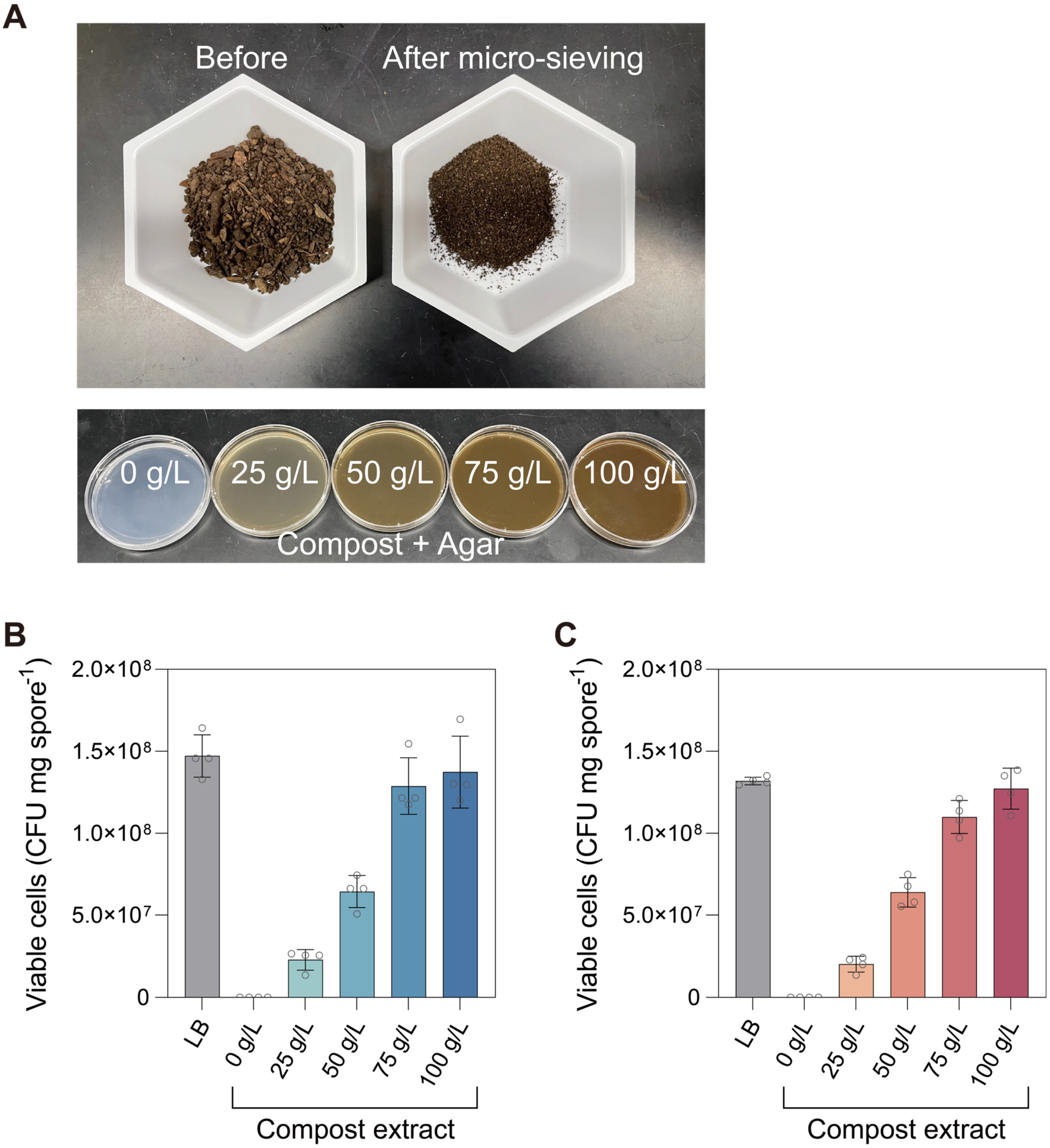

Supplementary Fig. 14. Spore germination on compost extract gels. (A) Preparation of compost extract gels to assess nutrient availability for spore germination. CFU assays with WT (B) and HST (C) spores on compost gel plates. Data are presented as mean values ± standard deviations from four independent experiments. Source data are provided as a Source Data file.

Supplementary Note 1: Spore germination on compost extract gels

To confirm *B. subtilis* spores can be germinated by utilizing nutrients in compost, CFU assay for ATCC 6633 WT and HST spores was carried out by using compost gel plates. Compost gel plates were prepared by the following procedure. Dried compost was sieved using a 35 standard mesh screen. The resulting compost powder was suspended in deionized water at various concentrations (25-100 g/L). Agarose powder was added to the compost suspension 15 g/L final concentration. The mixture solution was then autoclaved at 121 °C for 20 min. After autoclaving, the settled compost powder was separated, and the supernatant was carefully collected. The supernatant was aliquoted and solidified in petri-dishes.

CFU on LB gel plates served as positive controls. The WT and HST spores showed 1.47×10^8 and 1.32×10^8 CFU/mg, respectively, on nutrient-enriched LB plates (**Supplementary Fig. 14**). The CFU of spores on compost gel plates increased with the concentration of compost extract and showed up to 1.37×10^8 and 1.27×10^8 CFU mg^{-1}, respectively. These values corresponded to 93.3% and 96.4% germination efficiency compared to their positive controls, respectively. This result indicated that compost contains enough nutrients to trigger the spore germination, which is not surprising given the adaptability, fast growth rate, and widespread presence of *Bacillus* sp. in soil[1].

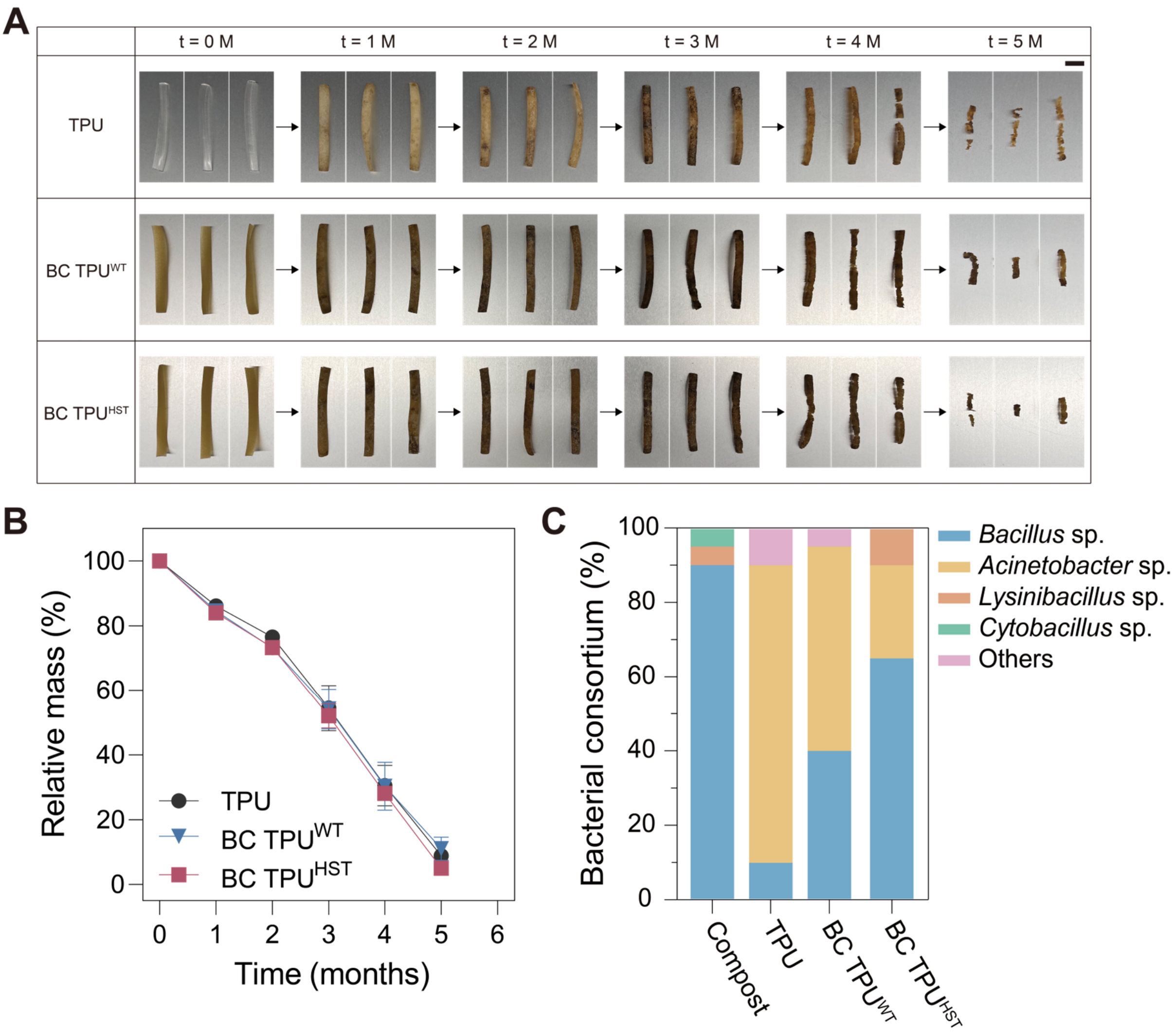

Supplementary Fig. 15. Gravimetric analysis of TPU degradation in untreated compost. Photographs depicting the visual changes observed in the disintegration (A) and mass loss profile (B) of TPU and BC TPUs during 5 months of incubation in untreated compost at 37 °C with 45-55% relative humidity (n = 3 per data point; *P < 0.05; **P < 0.01). Scale bar: 10 mm. Data are presented as mean values ± standard deviations from three independent experiments. (C) Bacterial consortium analyzed from the untreated compost and TPU surfaces incubated for 4 months in untreated compost at t = 4 months. Source data are provided as a Source Data file.

Supplementary Note 2: Disintegration of biocomposite TPU in untreated compost

After 5 months of incubation in a rich compost with confirmed microbial activity, TPU, BC TPU^{WT} and BC TPU^{HST} lost 91.1%, 89.0%, and 95.0% of their initial masses, respectively (**Supplementary Fig. 15A-B**). Even though all the TPU samples showed significant mass loss in compost, there was no notable difference in the disintegration rates among TPU and BC TPUs when other microbes were known to be present in the rich compost condition (i.e., a microbially active environment).

Sequencing analysis revealed that even though the degradation rates of TPU, BC TPU^{WT} and BC TPU^{HST} were similar, the bacterial consortium involved in the degradation of each TPU material was different (**Supplementary Fig. 15C**). *Acinetobacter* sp. in untreated compost was primarily responsible for the biodegradation of pristine TPU, but the portion of *Bacillus* sp. in the microbial consortium was significantly increased on BC TPU samples. It depicts that *B. subtilis* showed comparable disintegration activity toward TPU with the predominant TPU degrader in the compost, *Acinetobacter* sp.. The change of bacterial consortium by the introduction of *B. subtilis* spores into TPU is not surprising because *B. subtilis* is a well-known environmentally-friendly biocontrol agent, which competes with the pathogenic bacteria in soil and suppresses their growth[2]. The improved viability of HST spores, when compared to WT, after melt processing allowed *B. subtilis* to better compete against *Acinetobacter* sp. in compost.

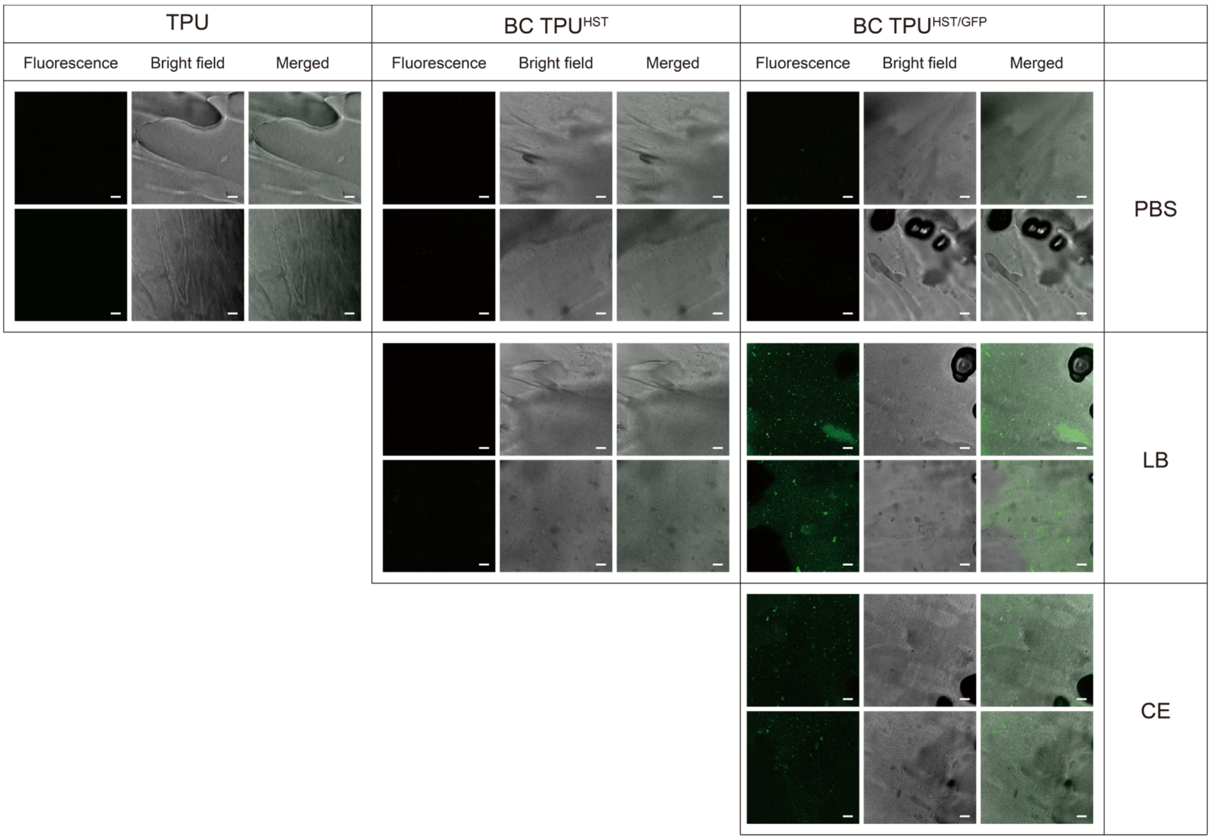

Supplementary Fig. 16. Additional CLSM images. Fluorescence, bright field and merged images (left to right) of TPU, BC TPU^{WT} and BC TPU^{HST} incubated in PBS, LB or CE obtained by CLSM (scale bars: 10 µm).

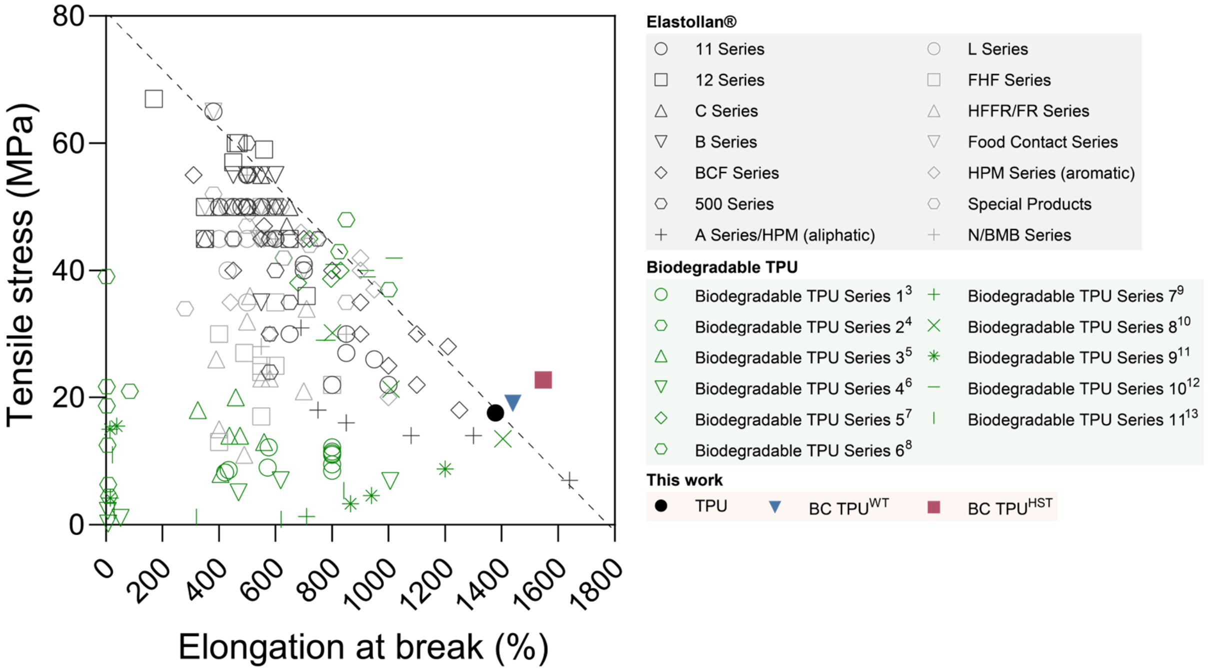

Supplementary Fig. 17. Tensile properties of commercial and biodegradable TPUs. Elongation at break and tensile stress of commercially-available TPUs (grayscale) were plotted with BC TPUs (colored) developed in this work. Previously reported biodegradable TPUs are plotted with green symbols[3–13]. Tensile properties of commercially-available TPUs were adapted from the BASF Elastollan® product range. The trade-off barrier (dashed line) was plotted by using the tensile properties of top 15 commercial TPUs that mutually exhibit high tensile stress and elongation at break. Source data are provided as a Source Data file.

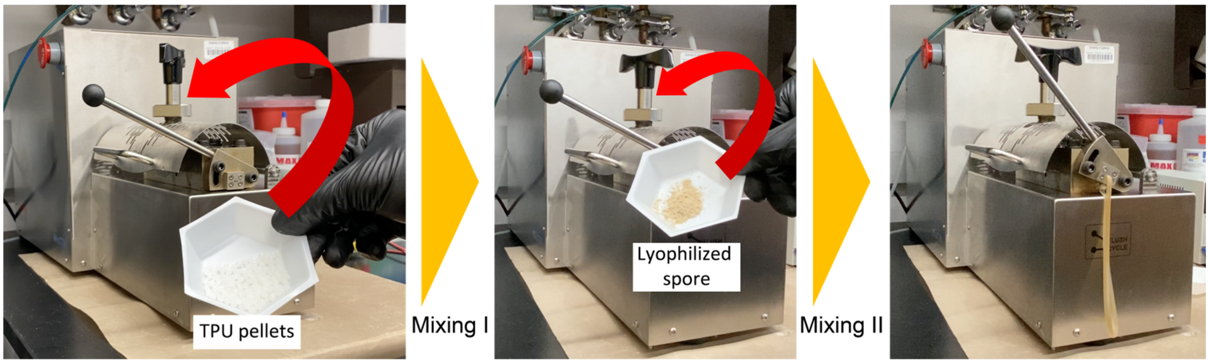

Supplementary Fig. 18. Biocomposite TPU fabrication using benchtop twin screw extruder. Fabrication of BC TPU^{HST} using benchtop twin screw extruder.

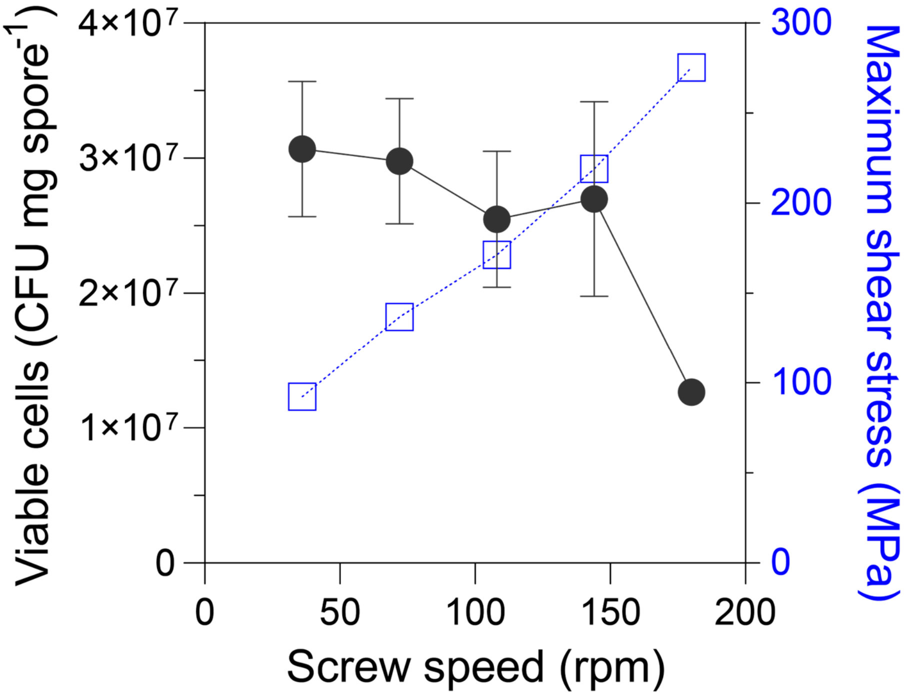

Supplementary Fig. 19. Spore viability and maximum shear stress at different screw speed. Viability of ATCC 6633 HST spores and maximum shear stress in the TSE at different screw speeds. Data are presented as mean values ± standard deviations from three independent experiments. Source data are provided as a Source Data file.

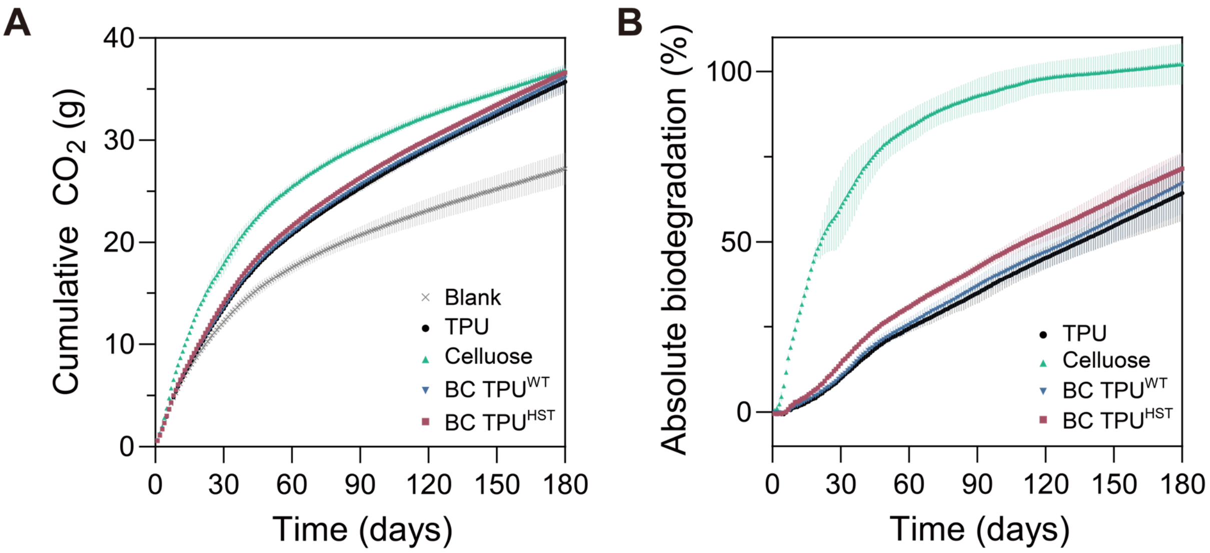

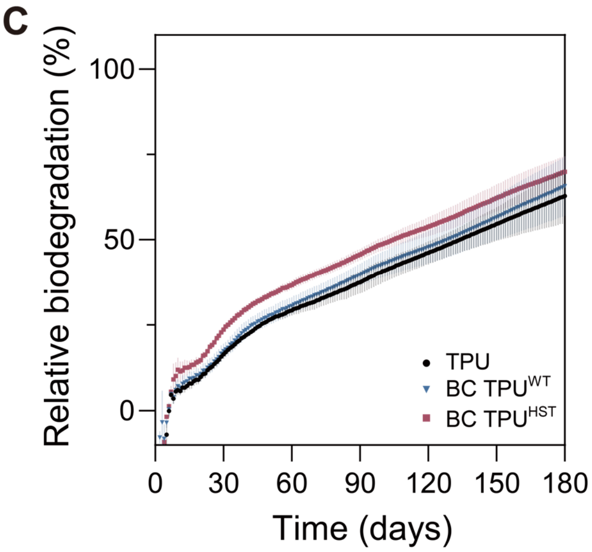

D

	Cumulative CO_2 (g)	Absolute biodegradation (%)	Relative biodegradation (%)
Blank	27.2	N/A	N/A
Cellulose	36.9	102.2	N/A
TPU	35.7	64.3	62.9
BC TPU^{WT}	36.1	67.2	65.7
BC TPU^{HST}	36.6	71.5	70.0

Supplementary Fig. 20. Respirometry analysis of TPU degradation in untreated compost. Cumulative CO_2 production (A), absolute biodegradation (B) and relative biodegradation (C) profiles of blank, cellulose, TPU, BC TPU^{WT} and BC TPU^{HST} in compost at 42 °C at 45–60% relative humidity. Data are presented as mean values ± standard deviations from three independent experiments. (D) 180-day averages of cumulative CO_2 production and percent biodegradation values for each test group. Relative biodegradation was calculated from the absolute biodegradation of samples relative to that of the reference material, cellulose. Source data are provided as a Source Data file.

Supplementary Note 3: Respirometry analysis of TPU degradation in untreated compost

TPU, BC TPU^{WT} and BC TPU^{HST} displayed similar mineralization by the end of testing with 35.7 g, 36.1 g and 36.6 g cumulative CO_2 production, respectively, after 180 days of incubation. Absolute biodegradation of TPU, BC TPU^{WT} and BC TPU^{HST} was calculated as 64.3%, 67.2% and 71.5%, respectively. Given that the absolute biodegradation of cellulose was 102.2%, relative biodegradation of TPU, BC TPU^{WT} and BC TPU^{HST} corresponded to 62.9%, 65.7% and 70.0%, respectively (**Supplementary Fig. 20**).

Supplementary Table 1. CFU assay of untreated and autoclaved compost. Number of viable cells in untreated and autoclaved compost. Cell viability of autoclaved compost was also tested after 5 M of incubation under 37 °C at 45-55 % relative humidity. Data are presented as mean values ± standard deviations from three independent experiments. Source data are provided as a Source Data file.

**Sample**	**Viable cells (CFU mg compost^{-1})**	**Cell survivability (%)**
Compost	1.6 (± 0.3) x 10^6	100
Autoclaved compost	2.6 (± 0.3)	0.0002
Autoclaved compost after 5 M of incubation	7.9 (± 1.0) x 10^3	0.5

Supplementary Table 2. Elemental analysis of compost. Elemental metal analysis of compost performed via total acid digestion.

Element	Recommended specification	Compost for gravimetric biodegradation	Compost for respirometry
Al (aluminum)	No limit	14700	11545
As (arsenic)	< 20 ppm	3.25	2.31
B (boron)	No limit	25.08	31.30
Ca (calcium)	No limit	15233	26901
Cd (cadmium)	< 2 ppm	0.18	0.88
Cr (chromium)	< 100 ppm	19.19	17.60
Cu (copper)	< 100 ppm	101.26	64.50
Fe (iron)	No limit	11677	11421
K (potassium)	No limit	6033	8758
Mg (magnesium)	No limit	2695	3910
Mn (manganese)	< 3500 ppm	532.9	613.0
Mo (molybdenum)	< 440 ppm	2.01	2.31
Na (sodium)	No limit	1154	1224
Ni (nickel)	< 50 ppm	7.76	4.79
P (phosphorus)	No limit	11578	10471
Pb (lead)	< 75 ppm	14.84	23.80
S (sulfur)	No limit	3413	2920
Total Solids (%)	50 – 55%	59.05	42.44
Volatile Solids (%)	N/A	23.63	17.60
Ash (%)	<70%	35.42	24.84
pH	7.0 – 8.2	7.02	7.40
Carbon (%)	N/A	20.28	27.04
Nitrogen (%)	N/A	1.74	2.49
C:N	10 – 40	11.66	10.86

Supplementary Table 3. Carbon and nitrogen content of test materials for respirometry. Carbon and nitrogen content of test materials and positive control for respirometry composting experiment. Limit of detection is 0.10%.

**Sample**	**Total Carbon (%)**	**Total Nitrogen (%)**
Cellulose	42.88	0.19
TPU	58.85	7.36
BC TPU^{WT}	58.76	7.28
BC TPU^{HST}	59.37	7.26

Supplementary References

1. Su, Y., Liu, C., Fang, H. & Zhang, D. *Bacillus subtilis*: a universal cell factory for industry, agriculture, biomaterials and medicine. *Microb. Cell Fact.* **19**, 173 (2020).
2. Morales-Cedeño, L. R. *et al.* Plant growth-promoting bacterial endophytes as biocontrol agents of pre- and post-harvest diseases: Fundamentals, methods of application and future perspectives. *Microbiol. Res.* **242**, 126612 (2021).
3. Tan, L., Su, Q., Zhang, S. & Huang, H. Preparing thermoplastic polyurethane/thermoplastic starch with high mechanical and biodegradable properties. *RSC Adv.* **5**, 80884–80892 (2015).
4. Ye, S.-H. *et al.* Nonthrombogenic, biodegradable elastomeric polyurethanes with variable sulfobetaine content. *ACS Appl. Mater. Interfaces* **6**, 22796–22806 (2014).
5. Guan, J., Sacks, M. S., Beckman, E. J. & Wagner, W. R. Biodegradable poly(ether ester urethane)urea elastomers based on poly(ether ester) triblock copolymers and putrescine: synthesis, characterization and cytocompatibility. *Biomaterials* **25**, 85–96 (2004).
6. Caracciolo, P. C., Buffa, F. & Abraham, G. A. Effect of the hard segment chemistry and structure on the thermal and mechanical properties of novel biomedical segmented poly(esterurethanes). *J. Mater. Sci. Mater. Med.* **20**, 145–155 (2009).
7. Fang, J. *et al.* Thiol click modification of cyclic disulfide containing biodegradable polyurethane urea elastomers. *Biomacromolecules* **16**, 1622–1633 (2015).
8. Yi, J. *et al.* Degradable polyurethane based on star-shaped polyester polyols (trimethylolpropane and ε-caprolactone) for marine antifouling. *Prog. Org. Coat.* **87**, 161–170 (2015).
9. Mi, H.-Y. *et al.* Biocompatible, degradable thermoplastic polyurethane based on polycaprolactone-block-polytetrahydrofuran-block-polycaprolactone copolymers for soft

tissue engineering. *J. Mater. Chem. B Mater. Biol. Med.* **5**, 4137–4151 (2017).

10. Wang, Z. *et al.* Fabrication and properties of a bio-based biodegradable thermoplastic polyurethane elastomer. *Polymers (Basel)* **11**, 1121 (2019).
11. Gokyer, S. *et al.* 3D printed biodegradable polyurethaneurea elastomer recapitulates skeletal muscle structure and function. *ACS Biomater. Sci. Eng.* **7**, 5189–5205 (2021).
12. Rajput, B. S. *et al.* Renewable low viscosity polyester-polyols for biodegradable thermoplastic polyurethanes. *J. Appl. Polym. Sci.* **139**, (2022).
13. Joo, H. *et al.* Functionalized thermoplastic polyurethane with tunable tribopolarity and biodegradability for high performance and biodegradable triboelectric nanogenerator. *Sustain. Mater. Technol.* **36**, e00638 (2023).
