# Supplementary material for: Biocomposite thermoplastic polyurethanes containing evolved bacterial spores as living fillers to facilitate polymer disintegration
Source: Nat Commun. 2024 Apr 30;15:3338. doi: 10.1038/s41467-024-47132-8 (PMC11061138; doi:10.1038/s41467-024-47132-8)
Supplement: Supplementary file 3 — Description of Additional Supplementary Files [file 41467_2024_47132_MOESM3_ESM.pdf]

Description of Additional of Supplementary Files

Supplementary Data 1. Whole genome sequencing of ALE-driven heat-shock tolerized strains.

Supplementary Movie 1. 3D X-ray microscopy of TPU.

Supplementary Movie 2. 3D X-ray microscopy of TPU bearing wild-type spores.

Supplementary Movie 3. 3D X-ray microscopy of TPU bearing heat-shock tolerized spores
