# Supplementary material for: Biocomposite thermoplastic polyurethanes containing evolved bacterial spores as living fillers to facilitate polymer disintegration
Source: Nat Commun. 2024 Apr 30;15:3338. doi: 10.1038/s41467-024-47132-8 (PMC11061138; doi:10.1038/s41467-024-47132-8)
Supplement: Supplementary file 8 — Reporting Summary [file 41467_2024_47132_MOESM8_ESM.pdf]

nature portfolio

Corresponding author(s): Md Arifur Rahman
Adam M. Feist
Jonathan K. Pokorski

Last updated by author(s): Feb 29, 2024

# Reporting Summary

Nature Portfolio wishes to improve the reproducibility of the work that we publish. This form provides structure for consistency and transparency in reporting. For further information on Nature Portfolio policies, see our Editorial Policies and the Editorial Policy Checklist.

## Statistics

For all statistical analyses, confirm that the following items are present in the figure legend, table legend, main text, or Methods section.

| n/a | Confirmed | |
|---|---|---|
| ☐ | ☒ | The exact sample size (*n*) for each experimental group/condition, given as a discrete number and unit of measurement |
| ☐ | ☒ | A statement on whether measurements were taken from distinct samples or whether the same sample was measured repeatedly |
| ☐ | ☒ | The statistical test(s) used AND whether they are one- or two-sided<br>*Only common tests should be described solely by name; describe more complex techniques in the Methods section.* |
| ☒ | ☐ | A description of all covariates tested |
| ☐ | ☒ | A description of any assumptions or corrections, such as tests of normality and adjustment for multiple comparisons |
| ☐ | ☒ | A full description of the statistical parameters including central tendency (e.g. means) or other basic estimates (e.g. regression coefficient) AND variation (e.g. standard deviation) or associated estimates of uncertainty (e.g. confidence intervals) |
| ☐ | ☒ | For null hypothesis testing, the test statistic (e.g. *F*, *t*, *r*) with confidence intervals, effect sizes, degrees of freedom and *P* value noted<br>*Give P values as exact values whenever suitable.* |
| ☒ | ☐ | For Bayesian analysis, information on the choice of priors and Markov chain Monte Carlo settings |
| ☒ | ☐ | For hierarchical and complex designs, identification of the appropriate level for tests and full reporting of outcomes |
| ☒ | ☐ | Estimates of effect sizes (e.g. Cohen's *d*, Pearson's *r*), indicating how they were calculated |

*Our web collection on statistics for biologists contains articles on many of the points above.*

## Software and code

Policy information about availability of computer code

Data collection

The following software were used for data collection:
(1) X-ray microscopy: Scout and Scan Control System (v12.0, Zeiss)
(2) Stress vs. Strain curves: Bluehill 3 (v3.24, Instron)
(3) Confocal laser scanning microscopy: LAS X (v1.4, Leica Microsystems)
(4) Scanning electron microscopy: xT microscope control (v13.9.1, Thermo Fisher Scientific)
(5) Spectrophotometry: Gen5 (v2.05, BioTek Instruments)
(6) Shear stress during hot melt extrusion: MiniCTW (v1.01, Thermo Fisher Scientific)
(7) Water contact angle: DROPimage Pro (v3.22, Ramé-hart Instrument co.)
(8) Gel permeation chromatography: LabSolutions (v5.103, Shimadzu Corporation)
(9) Attenuated total reflectance-Fourier transform infrared spectroscopy: OMNIC (v9.12, Thermo Fisher Scientific)
(10) Respirometry: Echo Software (ECHO Instruments)
(11) Elemental analysis: lyticOS® (Elementar)

| | |
|---|---|
| Data analysis | The following software were used for data analysis:<br>(1) RNA sequencing: Breseq (v0.35.4)<br>(2) X-ray microscopy: XM3DViewer (v1.2.6, Zeiss) and XMReconstructor (v10.6, Zeiss)<br>(3) Tensile properties: GraphPad Prism (v9.2.0, GraphPad)<br>(4) Confocal laser scanning microscopy: ImageJ (v2.14)<br>(5) Scanning electron microscopy: ImageJ (v2.14)<br>(6) Gel permeation chromatography: ASTRA (v8.0.0, Wyatt Technology)<br>(7) Statistical analysis: GraphPad Prism (v9.2.0, GraphPad) |

For manuscripts utilizing custom algorithms or software that are central to the research but not yet described in published literature, software must be made available to editors and reviewers. We strongly encourage code deposition in a community repository (e.g. GitHub). See the Nature Portfolio guidelines for submitting code & software for further information.

## Data

Policy information about availability of data

All manuscripts must include a data availability statement. This statement should provide the following information, where applicable:

- Accession codes, unique identifiers, or web links for publicly available datasets
- A description of any restrictions on data availability
- For clinical datasets or third party data, please ensure that the statement adheres to our policy

The raw resequencing data generated in this study have been deposited in the European Nucleotide Archive under accession code PRJNA981571 [https://www.ebi.ac.uk/ena/browser/view/PRJNA421359] and National Center for Biotechnology Information Sequence Read Archive under accession code PRJNA981571 [https://www.ncbi.nlm.nih.gov/bioproject/PRJNA981571/]. The variant calling data is available in ALEdb v1.088 under project name BS6633_HSTALE [https://aledb.org/ale/project/166/]. Source data are provided with this paper.

## Research involving human participants, their data, or biological material

Policy information about studies with human participants or human data. See also policy information about sex, gender (identity/presentation), and sexual orientation and race, ethnicity and racism.

| | |
|---|---|
| Reporting on sex and gender | N/A |
| Reporting on race, ethnicity, or other socially relevant groupings | N/A |
| Population characteristics | N/A |
| Recruitment | N/A |
| Ethics oversight | N/A |

Note that full information on the approval of the study protocol must also be provided in the manuscript.

# Field-specific reporting

Please select the one below that is the best fit for your research. If you are not sure, read the appropriate sections before making your selection.

☒ Life sciences ☐ Behavioural & social sciences ☐ Ecological, evolutionary & environmental sciences

For a reference copy of the document with all sections, see nature.com/documents/nr-reporting-summary-flat.pdf

# Life sciences study design

All studies must disclose on these points even when the disclosure is negative.

| | |
|---|---|
| Sample size | No sample size calculation was performed to predetermine sample size. Sample sizes (n ≥ 3) were chosen to ensure significant and reliable interpretation of the results based on previous experience with similar experiment types or standard practice in the field. |
| Data exclusions | No data were excluded from analyses. |
| Replication | All the main experimental findings were reproduced with at least three independent experiments. All attempts at replication were successful. |
| Randomization | This study does not contain any controlled trial to be randomized. |
| Blinding | This study does not contain any controlled trial to be blinded. |

# Reporting for specific materials, systems and methods

We require information from authors about some types of materials, experimental systems and methods used in many studies. Here, indicate whether each material, system or method listed is relevant to your study. If you are not sure if a list item applies to your research, read the appropriate section before selecting a response.

## Materials & experimental systems

| n/a | Involved in the study |
| --- | --- |
| ☒ | ☐ Antibodies |
| ☒ | ☐ Eukaryotic cell lines |
| ☒ | ☐ Palaeontology and archaeology |
| ☒ | ☐ Animals and other organisms |
| ☒ | ☐ Clinical data |
| ☒ | ☐ Dual use research of concern |
| ☒ | ☐ Plants |

## Methods

| n/a | Involved in the study |
| --- | --- |
| ☒ | ☐ ChIP-seq |
| ☒ | ☐ Flow cytometry |
| ☒ | ☐ MRI-based neuroimaging |

## Plants

| | |
| --- | --- |
| Seed stocks | N/A |
| Novel plant genotypes | N/A |
| Authentication | N/A |
